# Expression of the nociceptin/orphanin FQ receptor in the intestinal mucosa of IBS patients

**DOI:** 10.3892/etm.2013.1186

**Published:** 2013-06-27

**Authors:** LU LI, LEI DONG, SHENHAO WANG

**Affiliations:** Department of Gastroenterology, The Second Affiliated Hospital, Medical School of Xi’an Jiaotong University, Xi’an, Shaanxi 710004, P.R. China

**Keywords:** nociceptin/orphanin FQ, irritable bowel syndrome, intestinal mucosa

## Abstract

Nociceptin/orphanin FQ (N/OFQ) and the N/OFQ peptide (NOP) receptor play important roles in regulating gastrointestinal function. To assess whether the NOP receptor is implicated in the etiopathogenesis of irritable bowel syndrome (IBS), we measured the levels of NOP receptor mRNA and protein in the jejunal and colonic tissues of healthy subjects and patients with diarrhea-predominant IBS (D-IBS) and constipation-predominant IBS (C-IBS). Mucosal biopsies were obtained from the jejunum and colon of patients diagnosed with D-IBS and C-IBS by the Rome III criteria and from healthy control subjects. The expression of NOP receptor mRNA was measured quantitatively using quantitative PCR (qPCR) and NOP protein expression was assayed immunohistochemically using a rabbit monoclonal antibody to OFQ. NOP receptor mRNA was detected in the jejunum and colon of healthy subjects and was more highly expressed in the jejunum than in the colon. Expression was lower in the jejunum and colon of patients with D-IBS; however, it was similar in patients with C-IBS and healthy subjects. The numbers of OFQ-positive cells in the jejunum and colon were similar among the three groups. The NOP receptor may be involved in the regulation of intestinal movement in healthy individuals. Its involvement in the pathophysiology of IBS may depend on whether the IBS is constipation- or diarrhea-predominant.

## Introduction

Irritable bowel syndrome (IBS) is a chronic widespread disease responsible for 40% of outpatient consultations and it affects 15% of adults in western countries (1,2). IBS often has severe consequences, with patients often having impaired health-related quality of life (3–8). IBS is subcategorized into three types: constipation-predominant (C-IBS), diarrhea-predominant (D-IBS) and alternating diarrhea and constipation.

The pathophysiology of IBS may involve alterations in central processing, abnormal gastrointestinal motility and visceral hypersensitivity, and the interactions of these factors are possibly associated with the development of IBS symptoms. For example, the majority of patients with IBS have a lower pain threshold to colonic distension compared with that of healthy subjects (9).

Nociceptin/orphanin FQ (N/OFQ) and the N/OFQ peptide (NOP) receptor have been shown to be involved in the induction of vasodilation and the regulation of reward and motivation pathways related to substance abuse, with the NOP receptor being a candidate target for the treatment of obesity (10–12). N/OFQ and the NOP receptor are present in the central nervous system (CNS) and in the periphery, playing important roles in the modulation of gastrointestinal function and pain (13). To assess whether the NOP receptor is involved in the pathogeneses of IBS, we measured the levels of NOP receptor mRNA and protein in the jejunal and colonic tissues of healthy subjects and of patients with D-IBS and C-IBS.

## Subjects and methods

### Subjects

A total of 50 IBS patients who underwent endoscopic polypectomy were divided into the D-IBS group (27 cases) and the C-IBS group (23 cases) following diagnosis according to the Rome III criteria (14). Twenty healthy volunteers were selected as the control group. Subjects were excluded if they were <18 or >80 years of age, had a history of abdominal surgery, were unable to undergo enteroscopy under general anesthesia or colonoscopy, or had impaired blood coagulation function, including a platelet count <50×10^9^/ml or a bleeding time >14 min.

All subjects were informed about the purpose and methodology of the study and all provided written informed consent. The study protocol was approved by the ethics committee of Xi’an Jiaotong University (Xi’an, China).

### Specimens

Jejunal tissue biopsy specimens were obtained during double-balloon push enteroscopy and colon specimens were obtained during colonoscopy. Four specimens of jejunal mucosa 10 cm distal to the Treitz ligament were obtained from each subject who underwent enteroscopy and four specimens of colonic mucosa were obtained from the ascending colon 5 cm distal to the ileocecal valve of each subject who underwent colonoscopy.

Two jejunal mucosal and two colonic mucosal specimens from each subject were immediately frozen in liquid nitrogen (−170°C) and stored in an ultra-low refrigerator (−80°C). The remaining samples were fixed in 10% neutral formaldehyde solution and embedded in paraffin wax.

### RNA extraction

**Figure 1:**
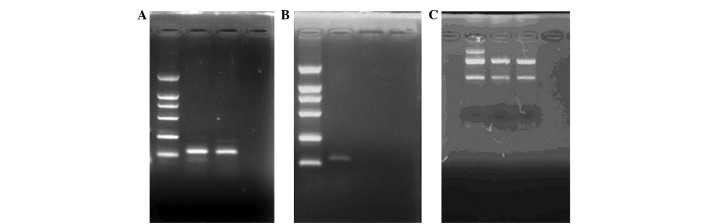
Agarose gel electrophoresis of qPCR amplification products. (A) Lane 1, DL2000 marker DNA (top to bottom: 2000, 1000, 750, 500, 240 and 100 bp); lane 2, β-actin (72 bp) and nociceptin/orphanin FQ peptide (NOP; 130 bp) mRNA; lane 3, NOP mRNA (130 bp). (B) Lane 1, DL2000 marker DNA; lane 2, NOP mRNA (130 bp). (C) Total RNA of the three specimens; note the 28S and 18S rRNA bands.

Total RNA was extracted from frozen specimens using TRIzol reagent (Invitrogen Life Technologies, Carlsbad, CA, USA) according to the manufacturer’s instructions and quantified by measurement of absorbance at 260 nm. RNA samples were evaluated using agarose gel electrophoresis, with the presence of 28S and 18S ribosomal RNA bands indicating the integrity of the samples (Fig. 1).

### Quantitative PCR (qPCR)

Following c-DNA synthesis, the expression of NOP receptor mRNA was assayed by amplification using the following primers: 5′-CTC GGC TGG TGC TGG TGG TA-3′ (forward) and 5′-CGT GCA GAA GCG CAG AAT GG-3′ (reverse). The expression was normalized relative to the expression of β-actin mRNA, which was amplified using the following primers: 5′-GGG TGT GAA CCA TGA GAA GTA TG-3′ (forward) and 5′-CCA TCAC GCC ACA GTT TCC-3′ (reverse). All primers were designed and synthesized by Sangon Biology Co. (Shanghai, China). Each 50 μl reaction contained 25 μl 2X PCR Master Mix (Roche Diagnostics, Switzerland), 1 μl each forward and reverse primer (final concentration, 0.2 μM each), 1 μl ROX (fluorescence base), 1 μl cDNA and 21 μl ddH_2_O. The amplification protocol consisted of 1 cycle of denaturation at 95°C for 2 min and 40 cycles of denaturation at 94°C for 30 sec, annealing at 62°C for 30 sec and extension at 72°C for 30 sec. The amplification products were assessed by electrophoresis in 1.6% agarose gels and staining in 0.5 μg/ml ethidium bromide.

**Figure 2:**
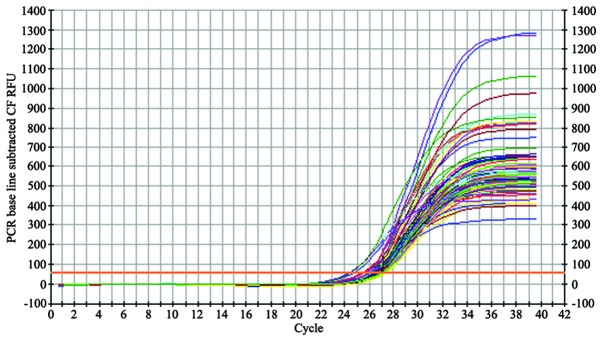
Fluorescence intensity curves for β-actin mRNA. PCR, polymerase chain reaction; CF, curve fit; RFU, relative fluorescence unit.

**Figure 3:**
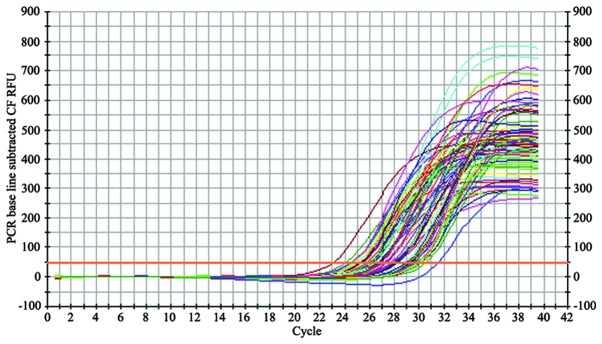
Fluorescence intensity curves for NOP receptor mRNA. PCR, polymerase chain reaction; CF, curve fit; RFU, relative fluorescence unit; NOP, nociceptin/orphanin FQ peptide.

Following each PCR amplification, the fluorescence intensity curve was generated automatically (Figs. 2 and 3). The fluorescence thresholds for β-actin and NOP receptor mRNA retrovirus product by the maximal curvature method were 44.2 and 56.4, respectively. The number of cycles at which the fluorescence signal reached the threshold was defined as the cycle threshold (Ct) value. To calibrate differences between each specimen and the retrovirus products, the β-actin Ct was subtracted from the NOP Ct value for each specimen. Standardized values were analyzed using the ΔΔCt method to determine the relative amount of NOP receptor mRNA in each specimen.

### Immunohistochemistry

Paraffin sections were dewaxed at room temperature using dimethylbenzene, twice for 15 min and then hydrated in an ethyl alcohol series (100, 95, 90, 80 and 70%) for 6 min. The samples were washed in 1X phosphate-buffered saline (PBS) for 35 min and the tissues were then blocked by incubation in 3% H_2_O_2_ for 15 min at 37°C. Then, the samples were washed three times for 1 min each in 1X PBS, incubated with goat serum for 15 min at 37°C and incubated with rabbit anti-nociceptin (1:200; Phoenix Pharmaceuticals, Inc., Burlingame, CA, USA) for 2–3 h at room temperature. The samples were washed three times for 5 min each with PBS and incubated with 50 μl goat anti-rabbit IgG-horseradish peroxidase (HRP) for 40 min at 37°C. Following three washes in PBS for 5 min each, the samples were incubated in HRP-labeled streptomycin-avidin working solution (S-A/HRP) for 30 min at 37°C. Then, the samples were washed three times for 5 min each with PBS and the color was developed using 3,3′-diaminobenzidine (DAB) for 1–2 min. The sections were rinsed in running water, duplicated with hematin, rinsed again in running water and dehydrated with laddered density alcohol. The samples were made transparent by incubation in dimethybenzene for 10 min, sealed with neutral tree glue and viewed by optical microscopy.

### Statistical analysis

All data are expressed as the mean ± standard error of the mean (SEM) and analyzed using the multi-independent sample Kruskal-Wallis H-test, with the Nemenyi test used to identify significant differences among the three groups. All statistical analyses were performed using SPSS 13.0 software (SPSS, Inc., Chicago, IL, USA). P<0.05 was considered to indicate a statistically significant difference.

## Results

### Demographic data

We assessed 20 healthy subjects as the control group, 27 patients diagnosed with D-IBS and 23 patients diagnosed with C-IBS. All subjects underwent colonoscopy or double-balloon pushed enteroscopy to obtain specimens of the colon or jejunum (Table I).

### Expression of NOP receptor mRNA

Using specific primers, we obtained a qPCR product for NOP mRNA of ~130 bp; the presence of 28S and 18S rRNA bands demonstrated that the total mRNA remained intact (Fig. 1). The fluorescence intensity curves for β-actin (Fig. 2) and the NOP receptor (Fig. 3) illustrate the quality of the mRNA samples.

### Expression of NOP receptor mRNA in the gut mucosa of healthy subjects

We observed that NOP receptor mRNA was expressed in all mucosal specimens from the control group, and the relative quantification was 7.86±4.66 in jejunum specimens and 1.04±0.33 in colon specimens respectively. This indicated that, the normalized level of expression was significantly higher in jejunum specimens than in colon specimens (Table II).

### Expression of NOP receptor mRNA in patients with IBS

The results showed that the relative expression of NOP receptor mRNA was 2.71±2.31 in jejunum specimens and 0.32±0.11 in colon specimens of D-IBS patients. These data were significantly lower than in samples from healthy controls. However, the relative expression of NOP receptor mRNA was 6.66±4.94 in jejunum specimens and 1.05±1.26 in colon specimens of C-IBS patients, and no difference was observed between C-IBS and the control (Table II).

### Immunohistochemistry

**Figure 4:**
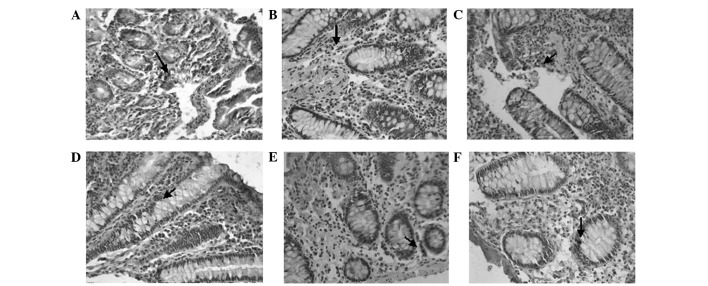
Anti-nociceptin antibody staining of jejunum specimens from the control (A), D-IBS (B) and C-IBS (C) groups, as well as staining of colon specimens from the control (D), D-IBS (E) and C-IBS (F) groups. Arrows indicate positive cells. D-IBS, diarrhea-predominant irritable bowel syndrome; C-IBS, constipation-predominant irritable bowel syndrome. Immuno-histochemical SABC staining; magnification, ×400.

The OFQ-positive cells were stained dark brown in color following incubation with anti-nociceptin antibody (Fig. 4). We observed no differences in the number of OFQ-positive cells among the control, D-IBS and C-IBS groups, in the colonic or jejunal mucosa.

## Discussion

The heptadecapeptide N/OFQ is the endogenous ligand for the NOP receptor and shares significant homology with classical opioid receptors (15,16). The N/OFQ system is widely distributed throughout the CNS and in peripheral organs of various species (15,17–21). Using qPCR, the N/OFQ system has been detected in the alimentary tracts of rats, pigs and guinea pigs (19,22). Using immunohistochemistry, N/OFQ has been shown to be localized in the rat enteric nervous system, with the mRNA encoding its precursor (prepro-OFQ/-N) and the cognate receptor ORL-1 expressed in the intestinal tract. Similar to classical opioids (23,24), N/OFQ has been shown to affect gastrointestinal motor and secretory responses, *in vitro* and *in vivo*. Unlike opioids, however, N/OFQ is insensitive to naloxone (25–27). Although N/OFQ has been observed to inhibit neurogenic contractions of the stomach and small intestine *in vitro*, it has also been shown to contract the rodent colon. *In vivo*, N/OFQ acts at sites in the central and peripheral nervous systems stimulating mechanical activity in the stomach and inhibiting this activity in the colon (25,28). Thus, N/OFQ acts as a neuromodulator of gastrointestinal motility and may have additional roles in the regulation of intestinal blood flow, active ion transport and immunity (25). Furthermore, the distribution and level of expression of the N/OFQ system differs among different species, as does the mechanism of regulation and functional sites (22–25).

In the current study, we demonstrated that NOP receptor mRNA and protein were present in the mucosal cells of the jejunum and colon of healthy subjects and patients with IBS, suggesting that the N/OFQ system plays an important role in regulating the pathophysiological onset of intestinal function. qPCR demonstrated that the level of expression of NOP receptor mRNA was higher in the jejunum than in the colon in healthy subjects, suggesting that the regulation of gastrointestinal motion, secretion and immunity is more complex in the jejunum than in the colon (29,30) and that the involvement of the NOP receptor in regulation is proportional to its level of expression.

Using immunohistochemical staining, we observed no differences in the number of OFQ-positive cells in the colonic or jejunal mucosa among the three groups of subjects, suggesting that OFQ may not be involved in the pathogenesis of IBS. These findings may also indicate, however, that the sensitivity of our immunohistochemical methods is insufficient to detect differences in protein expression (31,32).

The etiopathogenesis of IBS remains unclear. Although the majority of patients with IBS also have psychiatric symptoms, causality has not been demonstrated. Thus, although antidepressants are effective in the treatment of these patients, IBS is considered a functional gastrointestinal disease (33). Moreover, the pathogenesis of IBS is closely related to disturbances in the brain-gut axis (34).

The level of NOP mRNA in the colon and jejunum was lower in patients with D-IBS than in healthy subjects, whereas no differences were observed between patients with C-IBS and healthy subjects. It is not clear, however, whether these differences between D-IBS and C-IBS patients are related to the pathophysiological mechanisms of these types of IBS, whether an imbalance in the N/OFQ system is partly responsible for the oversensitivity of the intestinal tract in IBS patients or whether NOP overexpression (25,28) induces contraction of the human small intestine and colon in IBS patients.

In the central and peripheral nervous systems, the N/OFQ system regulates the release and transit of neurotransmitters, including norepinephrine, dopamine, 5-hydroxytryptamine and γ-aminobutyric acid (35,36). Our results suggest that the N/OFQ system also regulates the expression of neurotransmitters that act on intestinal motion and that the brain-gut axis is involved in these processes by an undetermined mechanism. Investigation of the N/OFQ system may be important in determining the pathogenesis of functional gastroenteropathies, including IBS.

## Figures and Tables

**Table I:** Demographic characteristics of the study subjects.

	Control		D-IBS		C-IBS	
			
	Jejunum	Colon	Jejunum	Colon	Jejunum	Colon
Number of subjects	9	11	15	12	10	13
Gender (male/female)	5/4	5/6	9/6	5/7	4/6	7/6
Age range (mean, years)	21–54 (34.0)	20–65 (34.0)	24–60 (36.0)	25–63 (37.2)	19–53 (34.4)	19–68 (38.3)
Course of disease (months)			45.4±24.6	52.8±29.3	89.7±62.8	82.1±56.4

D-IBS, diarrhea-predominant irritable bowel syndrome; C-IBS, constipation-predominant irritable bowel syndrome.

**Table II:** Relative quantification of NOP receptor mRNA in control subjects and IBS patients.

	Control		D-IBS		C-IBS	
			
	Jejunum	Colon	Jejunum	Colon	Jejunum	Colon
Number of subjects	9	11	15	12	10	13
Relative quantification	7.86±4.66	1.04±0.33^a^	2.71±2.31^b^	0.32±0.11^c^	6.66±4.94	1.05±1.26

Dara are presented as mean ± standard deviation.

a P<0.05, jejunum vs. colon of the control;

b P<0.05, jejunum of the D-IBS group vs. jejunum of the control;

c P<0.05, colon of the D-IBS group vs. colon of the control.

NOP, nociceptin/orphanin FG peptide; D-IBS, diarrhea-predominant irritable bowel syndrome; C-IBS, constipation-predominant irritable bowel syndrome.
